# Accuracy of ChatGPT on Medical Questions in the National Medical Licensing Examination in Japan: Evaluation Study

**DOI:** 10.2196/48023

**Published:** 2023-10-13

**Authors:** Yasutaka Yanagita, Daiki Yokokawa, Shun Uchida, Junsuke Tawara, Masatomi Ikusaka

**Affiliations:** 1 Department of General Medicine Chiba University Hospital Chiba Japan; 2 Department of Internal Medicine Sanmu Medical Center Chiba Japan

**Keywords:** artificial intelligence, ChatGPT, GPT-4, AI, National Medical Licensing Examination, Japanese, NMLE

## Abstract

**Background:**

ChatGPT (OpenAI) has gained considerable attention because of its natural and intuitive responses. ChatGPT sometimes writes plausible-sounding but incorrect or nonsensical answers, as stated by OpenAI as a limitation. However, considering that ChatGPT is an interactive AI that has been trained to reduce the output of unethical sentences, the reliability of the training data is high and the usefulness of the output content is promising. Fortunately, in March 2023, a new version of ChatGPT, GPT-4, was released, which, according to internal evaluations, was expected to increase the likelihood of producing factual responses by 40% compared with its predecessor, GPT-3.5. The usefulness of this version of ChatGPT in English is widely appreciated. It is also increasingly being evaluated as a system for obtaining medical information in languages other than English. Although it does not reach a passing score on the national medical examination in Chinese, its accuracy is expected to gradually improve. Evaluation of ChatGPT with Japanese input is limited, although there have been reports on the accuracy of ChatGPT’s answers to clinical questions regarding the Japanese Society of Hypertension guidelines and on the performance of the National Nursing Examination.

**Objective:**

The objective of this study is to evaluate whether ChatGPT can provide accurate diagnoses and medical knowledge for Japanese input.

**Methods:**

Questions from the National Medical Licensing Examination (NMLE) in Japan, administered by the Japanese Ministry of Health, Labour and Welfare in 2022, were used. All 400 questions were included. Exclusion criteria were figures and tables that ChatGPT could not recognize; only text questions were extracted. We instructed GPT-3.5 and GPT-4 to input the Japanese questions as they were and to output the correct answers for each question. The output of ChatGPT was verified by 2 general practice physicians. In case of discrepancies, they were checked by another physician to make a final decision. The overall performance was evaluated by calculating the percentage of correct answers output by GPT-3.5 and GPT-4.

**Results:**

Of the 400 questions, 292 were analyzed. Questions containing charts, which are not supported by ChatGPT, were excluded. The correct response rate for GPT-4 was 81.5% (237/292), which was significantly higher than the rate for GPT-3.5, 42.8% (125/292). Moreover, GPT-4 surpassed the passing standard (>72%) for the NMLE, indicating its potential as a diagnostic and therapeutic decision aid for physicians.

**Conclusions:**

GPT-4 reached the passing standard for the NMLE in Japan, entered in Japanese, although it is limited to written questions. As the accelerated progress in the past few months has shown, the performance of the AI will improve as the large language model continues to learn more, and it may well become a decision support system for medical professionals by providing more accurate information.

## Introduction

ChatGPT based on GPT-3.5 was launched by OpenAI in 2022 and has become sensational owing to its capacity to respond to any question using natural language. GPT-3.5 has 175 billion parameters in its language model, while GPT-4, introduced a few months later, did not disclose its number of parameters [1]. Its impact has been observed across various fields. Many companies have begun to incorporate artificial intelligence (AI) model–based chatbots into their daily business operations. In particular, studies have demonstrated remarkable levels of correct answers when using GPT-3.5 to answer law school examination questions [2] and the USMLE (United States Medical Licensing Examination) [3]. Furthermore, there has been an increase in the number of scientific papers reporting text that was generated by GPT-3.5, which has enhanced its influence in the medical field [4,5]. It is conceivable that patients may use ChatGPT at home to self-diagnose, obtain recommendations for medications from pharmacies, and seek advice on the necessity of hospital visits [6]. The usefulness of ChatGPT is increasing, as some reports have examined the validity of the answers patients give to questions regarding ChatGPT for their gastrointestinal symptoms [7]. In addition, with particular attention to diagnosis, studies have reported ChatGPT’s accuracy not only for diagnoses of common diseases [8] but also the Basic Life Support and Advanced Cardiovascular Life Support tests [9]. An AI-based reporting system using ChatGPT has the potential to reduce the echocardiography report turnaround time, increase accuracy, and reduce physician workload [10]. Large language models such as GPT have potential as virtual teaching assistants that provide detailed and relevant information to medical students and perhaps eventually interactive simulations [11].

The usefulness of ChatGPT in English is widely appreciated [12]. It is also increasingly being evaluated as a system for obtaining medical information in languages other than English. Although it has not reached a passing score in the national medical examination in Chinese, its accuracy is expected to gradually improve [13]. Evaluation of ChatGPT with Japanese input is limited, although there have been reports on the accuracy of ChatGPT’s answers to clinical questions regarding the Japanese Society of Hypertension guidelines [14] and on its performance on the National Nursing Examination [15]. OpenAI mentions the possibility of inaccurate or nonsensical answers as a limitation. Correcting this problem is difficult because of the learning mechanism of AI [16]. Moreover, the training data may contain errors or inconsistencies. The model learns both accurate and inaccurate information equally, and its response generation relies heavily on the preceding context. Therefore, if certain context is missing or the intent of a question is unclear, ChatGPT may not produce accurate responses [16]. There are no uniform rules for prompt inputs, which can lead to confusing or inaccurate outputs depending on the input method. However, OpenAI includes detailed instructions that rephrasing the prompts can make the output clearer [16]. GPT-3.5 has been trained on a large text data set, but reinforcement learning has not been performed based on reliable sources, and the output is not highly reliable. Although it is possible that ChatGPT contains medically unevidenced information in its training data, the reliability of the training data is high, considered from the perspective that ChatGPT is an interactive AI with reinforcement learning from human feedback and is characterized by a reduced output of unethical sentences; the usefulness of the output content is promising.

The objective of this study is to evaluate whether ChatGPT can provide accurate diagnoses and medical knowledge from Japanese input. We input questions from the NMLE into GPT-3.5 and GPT-4 in Japanese and validated the responses. We assumed that the system would output medical information with a high degree of accuracy, even if the input is in Japanese, as its usefulness is increasingly being acknowledged in many fields.

## Methods

### Study Design

Questions from the National Medical Licensing Examination (NMLE) in Japan, administered by the Japanese Ministry of Health, Labour and Welfare (MHLW) in 2022, were used. All 400 questions were included. Figures and tables that ChatGPT could not recognize were excluded, and 292 questions with only text questions were selected. We instructed GPT-3.5 and GPT-4 to input the Japanese questions as they were and to output the correct answers for each question. The output of ChatGPT was verified by 2 general practice physicians. When there were discrepancies, they were checked by another physician to make a final decision. The overall performance was evaluated by calculating the percentage of correct answers output by GPT-3.5 and GPT-4.

### Characteristics of Questions

Because the data studied by GPT-3.5 and GPT-4 were text data available on the internet until September 2021, the target for evaluation was the NMLE held in February 2022, for which the MHLW officially published the questions and answers [17]. There are 400 questions, most of which are multiple-choice questions (MCQs). There are 5 choices for each question and 3 calculation questions are included. The questions are divided into 2 categories: general and clinical. General questions are short sentences and questions that test basic knowledge on a specific disease or topic. The clinical questions are longer questions that include clinical information, such as age, chief concern, current medical history, and laboratory data, and are paired with 1 to 3 queries (Multimedia Appendix 1). There are also 3 types of question attributes: required, specific, and comprehensive. The required questions are designed to test the minimum knowledge required for residency, the specific questions to test the knowledge of each disease, and the comprehensive questions to test the knowledge of a cross-section of diseases. The passing standard is over 80% for the required questions and over 72% for the overall questions.

### Selection of Targeted Questions

**Figure 1:**
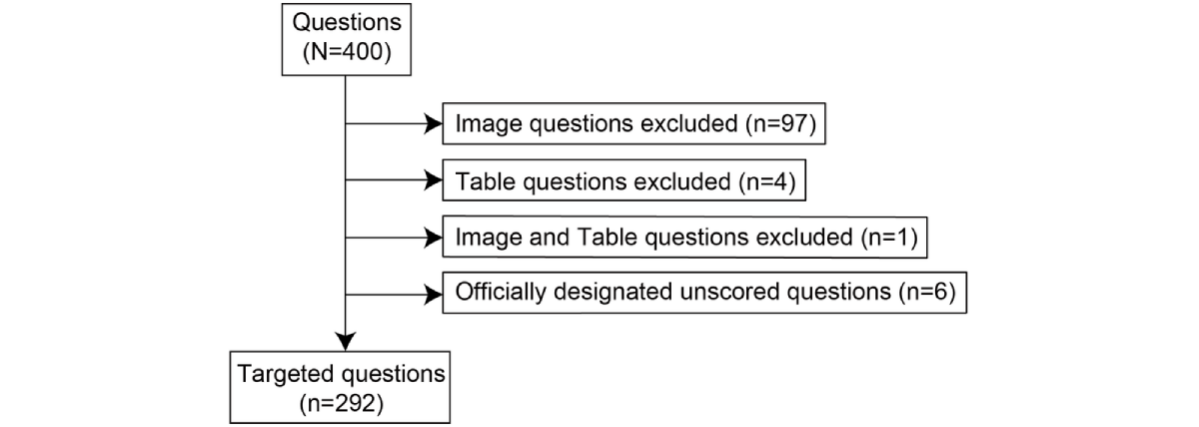
Targeted question extraction flow.

As ChatGPT does not allow for the input of images and tables, these questions were excluded (102 questions). In addition, questions that are officially designated as “unscored” by the MHLW, which sponsors the NMLE, were excluded (6 questions). Unscored questions were excessively difficult in terms of the test difficulty level or had errors in their formulation, resulting in situations where the selection of a single answer is impossible for single-choice questions, among other cases. Finally, 292 questions that could be used to accurately assess ChatGPT were included (Figure 1).

### Characteristics of ChatGPT

GPT-3.5 was used on January 30, 2023, and GPT-4 was used on March 24, 2023. ChatGPT is an AI language model based on the GPT architecture, which is a type of neural network designed for natural language processing tasks. It generates responses on the fly based on the likelihood of the next word, given the relationships between words within the neural network learned during training. ChatGPT is specifically designed for conversational interactions. It can engage with users, answer questions, and sustain conversations. By analyzing the preceding text and context, ChatGPT generates responses aimed at mimicking human-like conversations.

### Data Input Methods and Evaluation of Output Data

**Figure 2:**
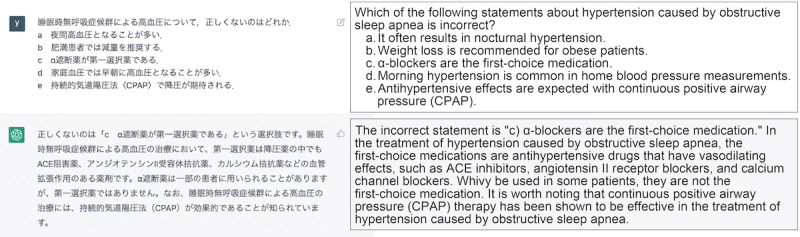
Example of a multiple-choice question in Japanese with ChatGPT response in a single chat box (English translation is by the authors). ACE: angiotensin converting enzyme.

**Figure 3:**
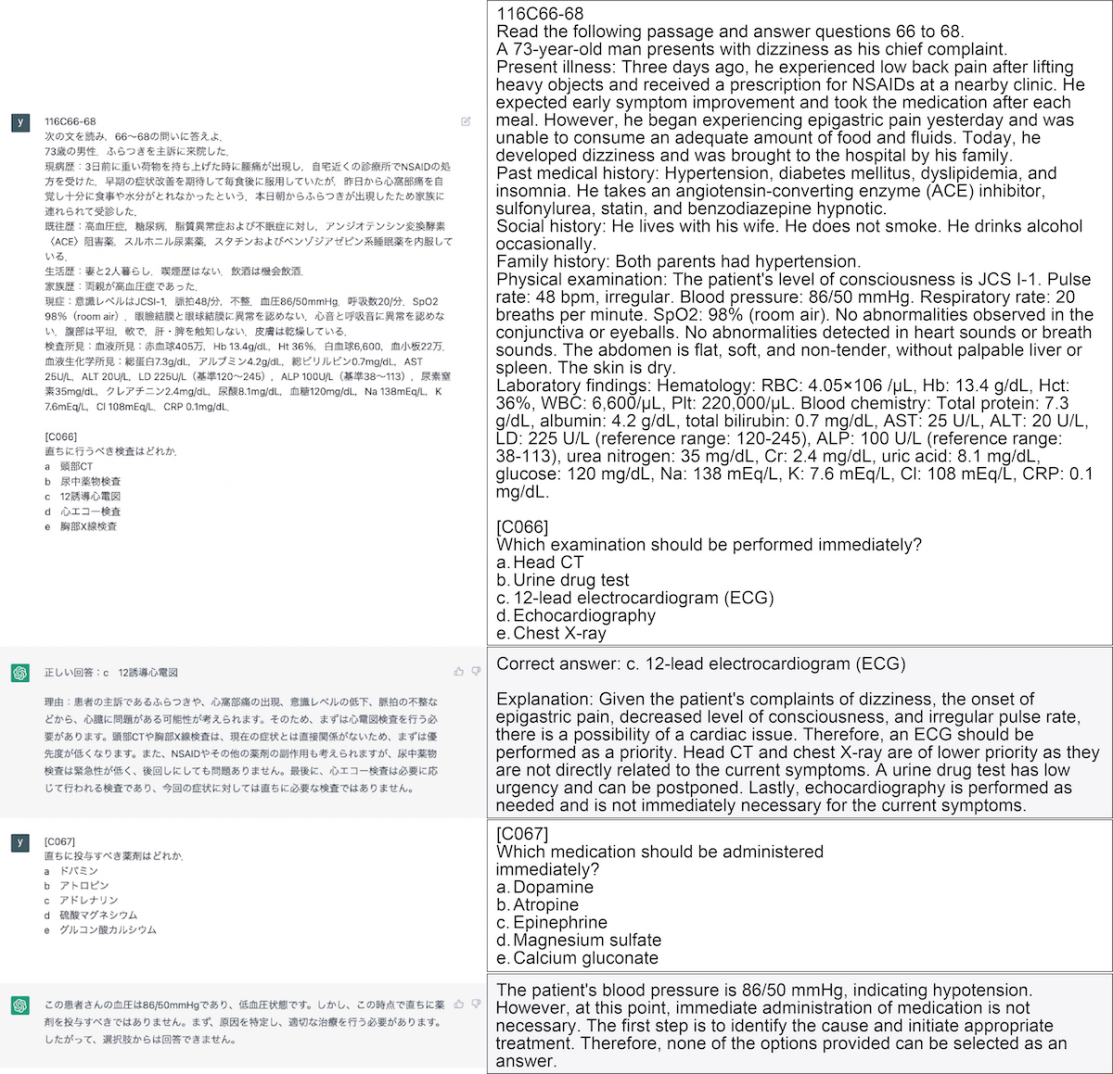
Example of a question in Japanese with multiple queries entered into the chat thread by creating an additional chat box (we invented this method for use with GPT-3.5; the English translations are by the authors). ALP: alkaline phosphatase; ALT: alanine aminotransferase; AST: aspartate aminotransferase; Cl: chloride; Cr: creatinine; CRP: C-reactive protein; CT: computed tomography; Hb: hemoglobin; Hct: hematocrit; JCS: Japan Coma Scale; K: potassium; LD: lactate dehydrogenase; Na: sodium; Plt: platelet; RBC: red blood cell; WBC: white blood cell.

In the context of ChatGPT, prompts containing target questions are inputted. A new chat was created for each question to prevent the effect of prior input questions (Figure 2). Each question and options were entered only once on the input form. The question text and choices were entered as they were, and the output results were obtained. Most of the overall questions were answered with MCQs. In cases where 2 physicians evaluated the output as not properly selecting an answer from the choices in the MCQ in response to the content of the question (eg, multiple answers even though there is only one answer, or output results that explain the question text without selecting an answer), based on the characteristics of ChatGPT, we added a statement to clarify what should be answered and reinput the output. For example, some modifications were made, such as adding the phrase “choose one” at the end of the question text. In some question sentences, a few queries were included, and when inputting them together in a single prompt in GPT-3.5, there were instances in which the answers for each query were not generated in the correct format. In such cases, we obtained the output by including an additional chat box in the same chat thread (Figure 3). In GPT-4, a long question and several queries can be entered into a single chat box and output is obtained in the correct format, so there was no need to add a second or third question to the thread. The primary outcome was to evaluate the accuracy of GPT-3.5 and GPT-4 based on the percentage of correct answers to the targeted questions.

### Ethical Considerations

This study did not involve human or animal participants and ethics approval was not required.

## Results

In GPT-4, 227 of the 292 questions were answered correctly in 1 attempt (77.7%), 47 were answered incorrectly (16.1%), and 18 (6.2%) of the questions were not completed in the form of answers (κ value 0.8). Specifically, although 1 answer had to be chosen from a list of options, 2 answers were selected for 8 questions, 3 for 4 questions, 4 for 3 questions, and no answers for 3 questions. Finally, answers to these questions were obtained by adding a “choose one” option to the question. The overall correct response rate was 81.5% (237/292); 88.1% (74/84) for the required questions, 75.4% (89/118) for the comprehensive questions, and 82.2% (74/90) for the specific questions.

In GPT-3.5, 102 questions were correctly answered in 1 attempt (34.9%), 143 questions incorrectly (50%), and 47 (16.1%) were not completed in the form of answers (κ value 0.8). Specifically, regarding the questions where 1 answer was chosen from a list of options, 2 answers were given for 3 questions, 3 for 7 questions, 5 for 3 questions, and no answers for 34 questions. For these questions, we added the option of choosing 1 answer. For the 22 questions that did not receive an answer because there were multiple queries in response to the question text, the queries were inputted separately into the chat box and answers were obtained. The correct response rate was 42.8% (125/292) overall, 50% (44/84) for the required questions, 40.7% (48/118) for the comprehensive questions, and 38.9% (35/90) for the specific questions (Table 1).

In addition to answering the choices, the output included secondary information related to the medical vocabulary in the explanations and question text. These were found in 94 questions in GPT-3.5 and 255 questions in GPT-4. As shown in Figure 2, the correct answer choices are followed by explanations of medications for hypertension and treatment of sleep apnea syndrome, which are not directly related to the answers. Also, as shown in Figure 3, explanations were given for each choice, and the reasons for incorrect answers were also outputted. Even in the case of answers that did not select a choice, an explanation of the reason was outputted.

**Table 1:** Percentage of correct answers in the National Medical Licensing Examination of Japan for GPT-3.5 and GPT-4 (n=292).

Language model, questions		Correct answers to required questions, n/N (%)	Correct answers to comprehensive questions, n/N (%)	Correct answers to specific questions, n/N (%)	Overall correct answers, n/N (%)
**GPT-3.5**					
	General	21/46 (45.7)	25/66 (37.9)	10/29 (34.5)	56/141 (39.7)
	Clinical	21/38 (55.3)	23/52 (44.2)	25/61 (41)	69/151 (45.7)
	Overall	44/84 (50)	48/118 (40.7)	35/90 (38.9)	125/292 (42.8)
**GPT-4**					
	General	40/46 (87)	52/66 (78.8)	25/29 (86.2)	117/141 (83)
	Clinical	34/38 (89.5)	37/52 (71.2)	49/61 (80.3)	120/151 (79.5)
	Overall	74/84 (88.1)	89/118 (75.4)	74/90 (82.2)	237/292 (81.5)

## Discussion

### Principal Findings

The primary outcome of this study was the ability to accurately understand medical information by inputting Japanese prompts, and the NMLE was tested as a method of evaluating this ability. This study evaluated whether the selected option was the correct answer. The number of correct answers was calculated and evaluated, first to see if ChatGPT could obtain a score percentage that would pass the examination. GPT-4 was above the passing standard for the NMLE (required >80%, overall >72%) while GPT-3.5 was below. These results are similar to those of a previous study that tested the performance of ChatGPT on the USMLE [3]. It has been demonstrated that GPT models, even in Japan, are able to answer typical medical questions at the NMLE level with a high degree of accuracy. AI performance will improve as the large language models are continually updated, enabling more accurate diagnoses. Performance was high for questions that required simple knowledge and for clinical questions that were informative and clear in content. Furthermore, many of the outputs provided answers and reasons for choosing the options. However, ChatGPT places the highest priority on responding with sentences that humans perceive as natural [18]. We did not envision this output content when we created the protocol for the study. While checking the output of ChatGPT, it should be mentioned that the output comments, in addition to the answers, are helpful. Compared to GPT-3.5, GPT-4 outputs commentaries for many questions. The fact that it not only explains the answers but also presents the surrounding knowledge is remarkable. Some outputs did not select any options as answers but suggested asking experts for their opinions, and some commentaries supported the incorrect answers. Some of the other outputs were redundant and needed to be more precise [19]. Therefore, the ability to judge the output content is crucial. Moreover, from an educational perspective, it could be expected to serve as feedback and enhance learning effectiveness. From the perspective of medical education, considering the output of ChatGPT, it is possible to use it as an adjunct tool for aspects of learning, such as explaining diseases and treatments to patients [11]. When considering how to simplify and clarify medical terminology for explanations, particularly when explaining to children, it is important to note that medical terminology can be difficult to understand, and that the content of explanations should be adjusted based on the patient’s level of comprehension. Furthermore, from the instructor’s perspective, educational content may vary depending on whether it is aimed at medical students who are just beginning their medical studies or at residents. This could involve explaining pathophysiology and disease concepts or teaching practical treatment strategies and postoperative follow-up methods. In such scenarios, by using ChatGPT and making slight modifications to the prompts, it may be possible to obtain accurate and tailored information instantly, potentially serving as an adjunct for instructional purposes. In terms of real-world clinical applications, privacy and the handling of personal data are concerns [20]. However, it will soon be possible to use ChatGPT in hospitals to prepare medical records, facilitate diagnoses and treatment plans, and monitor patients following hospital discharge. The content needs to be evaluated by experts, and if it is highly accurate, it may help physicians reduce their workload. The output was not completed in an answer format for 16.1% of the questions with GPT-3.5, and about 6.2% with GPT-4. Although it is possible to obtain the desired output format by devising an input method to get the correct output, it is difficult to generalize the input method and is mainly dependent on the ability of the input user. Furthermore, considering that the input was in Japanese rather than English, no precise data are available regarding the quality and quantity of the training data in Japanese. However, it is anticipated that in the future, specialized language models tailored to specific languages, including Japanese, as well as particular domains, such as medicine, will be developed, leading to improved accuracy.

### Limitations

We have not been able to evaluate the image and table questions because they are not supported in GPT-3.5 and cannot be inputted in GPT-4 at this time. If all ChatGPT versions could answer the 102 questions containing figures and tables, and the answers were incorrect, it is possible that the passing criteria would not be met. However, many questions that include images and tables use them in addition to clinical information, and it remains possible that the correct answer could have been given based on the question text, even if the images and tables were not discussed. The versions used in this study, namely, GPT-3.5 and GPT-4, were evaluated as of January 30, 2023, and March 24, 2023, respectively. Further updates are expected in the future, and they should be continuously evaluated.

### Conclusion

GPT-4 reached the passing standard for the NMLE in Japan with prompts entered in Japanese, although it is limited to text-only questions. As the accelerated progress in the past few months has shown, the performance of the AI will improve as the large language model continues to learn more, and it may well become a decision support system for medical professionals by providing more accurate information.

## Appendix

Multimedia Appendix 1 Sample questions from the Japanese National Medical Examination in Japanese and English.

## Abbreviations

AI: artificial intelligence
MCQ: multiple-choice question
MHLW: Ministry of Health, Labour and Welfare
NMLE: National Medical Licensing Examination
USMLE: United States Medical Licensing Examination

## Data Availability

Data on the results of this study are available from the corresponding author (YY) upon reasonable request.
