# Supplementary material for: Accuracy of ChatGPT on Medical Questions in the National Medical Licensing Examination in Japan: Evaluation Study
Source: JMIR Form Res. 2023 Oct 13;7:e48023. doi: 10.2196/48023 (PMC10612006; doi:10.2196/48023)
Supplement: Multimedia Appendix 1 [file formative_v7i1e48023_app1.docx]

Supplement 1: Sample questions from the National Medical Licensing Examination in Japanese and English.

1. General question

| 睡眠時無呼吸症候群による高血圧について，正しくないのはどれか．  a　夜間高血圧となることが多い．  b　肥満患者では減量を推奨する．  c　α遮断薬が第一選択薬である．  d　家庭血圧では早朝に高血圧となることが多い．  e　持続的気道陽圧法〈CPAP〉で降圧が期待される． | Which of the following statements about hypertension caused by obstructive sleep apnea is incorrect?  a. It often leads to nocturnal hypertension.  b. Weight loss is recommended for obese patients.  c. Alpha-blockers are the first-choice medication.  d. Morning hypertension is common in home blood pressure measurements.  e. Continuous positive airway pressure (CPAP) therapy is expected to reduce blood pressure. |
| --- | --- |

2. Clinical question

| 次の文を読み，72〜74の問いに答えよ．  87歳の女性．両側下腿浮腫を主訴に夫と息子夫婦とともに来院した．  現病歴：約半年前から両側下腿浮腫が出現し，自宅近くの医療機関で少量の利尿薬を処方されていた．3ヵ月前から下腿浮腫の悪化のため歩きにくくなり，労作時の息切れも感じるようになった．2週間前からは食欲低下も著しくなったため受診した．既往歴：下腿浮腫に対して少量のループ利尿薬を処方されている．  生活歴：夫（94歳）と2人暮らし．喫煙歴と飲酒歴はない．  家族歴：妹が悪性リンパ腫であった．  現症：意識は清明．身長153cm，体重45kg．体温36.7℃．脈拍72/分，整．血圧152/70mmHg．呼吸数16/分．SpO2 98％（room air）．眼瞼結膜は貧血様である．頸静脈の怒張を認める．心音はLevine 2/6の全収縮期雑音を認める．呼吸音に異常を認めない．腹部は肋骨弓下に肝臓を1cm触知する．下腿浮腫を両側に認めるが熱感や圧痛はなく，圧迫により生じる圧痕は圧迫を解除すると戻る．四肢に明らかな麻痺を認めない．  検査所見：尿所見：蛋白（－），糖（－），潜血（－）．血液所見：赤血球350万，Hb 8.1g/dL，Ht 27％，白血球7,800．血液生化学所見：総蛋白5.1g/dL，アルブミン2.5g/dL，AST 40U/L，ALT 50U/L，LD 282U/L（基準120〜245），CK 70U/L（基準30〜140），尿素窒素19mg/dL，クレアチニン1.0mg/dL，血糖93mg/dL，HbA1c 5.6％（基準4.6〜6.2），総コレステロール160mg/dL，トリグリセリド166mg/dL，Na 142mEq/L，K 3.8mEq/L，Cl 108mEq/L，Ca 7.5mg/dL，Fe 10μg/dL，TSH 3.6（基準0.2〜4.0），FT3 2.4（基準2.3〜4.3），FT4 0.9（基準0.8〜2.2），BNP 110pg/mL（基準18.4以下）．CRP 0.2mg/dL．12誘導心電図で完全右脚ブロックを認める．胸部X線写真で心  胸郭比50％，肺血管影の増強はなく，両側の肋骨横隔膜角の鈍化を認める．  問72浮腫の原因で可能性が高いのはどれか．2つ選べ．  a　腎性  b　心性  c　炎症性  d　内分泌性  e　低蛋白性  問73貧血の原因精査のために便潜血検査を行うことにした．  自宅での検体採取に関する患者への説明で適切なのはどれか．  a　「この検査は5日間連続で行います」  b　「できるだけ多く採取してください」  c　「容器に入れた便は室温で1週間保存可能です」  d　「採取前日の20時以降は食事をしないでください」  e　「1回の排便につき1つの容器に採取してください」  問74便潜血検査の結果が陽性であったため下部消化管内視鏡検査を行ったところ大腸癌と診断された．入院して腹腔鏡下手術を行い，術後経過は順調で退院予定である．退院前に高齢者総合機能評価〈CGA〉を行った．  評価すべきこととして適切でないのはどれか．  a　聴力  b　認知機能  c　運動機能  d　気分・意欲  e　基本的日常生活動作〈ADL〉 | Read the following passage and answer questions 72-74.  An 87-year-old woman presented to the clinic with her husband and son's family, complaining of bilateral lower leg edema.  Present illness: Bilateral lower leg edema appeared approximately six months ago, and she was prescribed a small dose of loop diuretic at a local medical facility near her home. Over the past three months, her lower leg edema worsened, making it difficult for her to walk, and she also experienced dyspnea on exertion. Two weeks ago, her appetite significantly decreased, leading her to seek medical attention. Past medical history: She has been prescribed a small dose of loop diuretic for lower leg edema. Social history: She lives with her 94-year-old husband. She has no history of smoking or alcohol consumption.  Family history: Her sister had malignant lymphoma.  Present illness: The patient is alert and oriented. She is 153 cm tall and weighs 45 kg. Her body temperature is 36.7℃. Pulse rate is 72 bpm and regular. Blood pressure is 152/70 mmHg. Respiratory rate is 16 breaths per minute. SpO2 is 98% on room air. Conjunctiva appears anemic. Jugular vein distention is observed. Cardiac auscultation reveals a grade 2/6 holosystolic murmur. No abnormal breath sounds are heard. On abdominal examination, the liver is palpable 1 cm below the costal margin. Bilateral lower leg edema is present, but there is no warmth, tenderness, or indentation caused by pressure, which returns to normal when pressure is released. No obvious paralysis is observed in the limbs. Laboratory findings: Urinalysis: Protein (-), glucose (-), occult blood (-). Complete blood count: Red blood cells 3.5 million, Hb 8.1 g/dL, Hct 27%, white blood cells 7,800. Biochemical analysis: Total protein 5.1 g/dL, Total protein 5.1 g/dL, albumin 2.5 g/dL, AST 40 U/L, ALT 50 U/L, LD 282 U/L (reference range 120-245), CK 70 U/L (reference range 30-140), blood urea nitrogen 19 mg/dL, creatinine 1.0 mg/dL, blood glucose 93 mg/dL, HbA1c 5.6% (reference range 4.6-6.2), total cholesterol 160 mg/dL, triglycerides 166 mg/dL, Na 142 mEq/L, K 3.8 mEq/L, Cl 108 mEq/L, Ca 7.5 mg/dL, Fe 10 μg/dL, TSH 3.6 (reference range 0.2-4.0), FT3 2.4 (reference range 2.3-4.3), FT4 0.9 (reference range 0.8-2.2), BNP 110 pg/mL (reference range ≤18.4), CRP 0.2 mg/dL. A 12-lead electrocardiogram shows complete right bundle branch block. Chest X-ray shows a cardiothoracic ratio of 50%, no enhancement of the pulmonary vasculature, and blunting of the costophrenic angles bilaterally.  Question 72:  Which of the following is most likely the cause of the edema? Choose two.  a) Renal  b) Cardiac  c) Inflammatory  d) Endocrine  e) Hypoproteinemic  Question 73:  To investigate the cause of anemia, the patient will undergo fecal occult blood testing. Which explanation about collecting the sample at home is appropriate?  a) "This test will be conducted continuously for five days."  b) "Please collect as much as possible."  c) "The collected stool can be stored at room temperature for one week."  d) "Please refrain from eating after 8:00 PM on the day before collection."  e) "Collect the sample in one container per bowel movement."  Question 74:  Due to a positive fecal occult blood test, lower gastrointestinal endoscopy was performed, and the patient was diagnosed with colorectal cancer. She was admitted and underwent laparoscopic surgery, and her postoperative course has been uneventful. Before discharge, a comprehensive geriatric assessment (CGA) was conducted.  Which aspect is not appropriate for evaluation?  a) Hearing  b) Cognitive function  c) Motor function  d) Mood and motivation  e) Activities of daily living (ADL) |
| --- | --- |
